# Removal of AMR plasmids using a mobile, broad host-range CRISPR-Cas9 delivery tool

**DOI:** 10.1099/mic.0.001334

**Published:** 2023-05-25

**Authors:** David Sünderhauf, Uli Klümper, Elizabeth Pursey, Edze R. Westra, William H. Gaze, Stineke van Houte

**Affiliations:** 1 Centre for Ecology and Conservation, University of Exeter, Environment and Sustainability Institute, Penryn, TR10 9FE, UK; 2 Institute of Hydrobiology, Technische Universität Dresden, 01217 Dresden, Germany; 3 European Centre for Environment and Human Health, University of Exeter Medical School, Environment and Sustainability Institute, Penryn, TR10 9FE, UK

**Keywords:** antimicrobial resistance, AMR gene removal, antibiotic resensitization, plasmid curing, CRISPR-Cas plasmids, broad host-range plasmids

## Abstract

Antimicrobial resistance (AMR) genes are widely disseminated on plasmids. Therefore, interventions aimed at blocking plasmid uptake and transfer may curb the spread of AMR. Previous studies have used CRISPR-Cas-based technology to remove plasmids encoding AMR genes from target bacteria, using either phage- or plasmid-based delivery vehicles that typically have narrow host ranges. To make this technology feasible for removal of AMR plasmids from multiple members of complex microbial communities, an efficient, broad host-range delivery vehicle is needed. We engineered the broad host-range IncP1-plasmid pKJK5 to encode *cas9* programmed to target an AMR gene. We demonstrate that the resulting plasmid pKJK5::csg has the ability to block the uptake of AMR plasmids and to remove resident plasmids from *Escherichia coli*. Furthermore, due to its broad host range, pKJK5::csg successfully blocked AMR plasmid uptake in a range of environmental, pig- and human-associated coliform isolates, as well as in isolates of two species of *Pseudomonas*. This study firmly establishes pKJK5::csg as a promising broad host-range CRISPR-Cas9 delivery tool for AMR plasmid removal, which has the potential to be applied in complex microbial communities to remove AMR genes from a broad range of bacterial species.

## Graphical Abstract

A broad host-range CRISPR-Cas9 delivery tool was developed and used to conjugatively remove antimicrobial resistance (AMR) plasmids (top) and protect a series of coliform and *Pseudomonas* isolates from AMR plasmid uptake (bottom).

## Introduction

Antimicrobial resistance (AMR) is one of the key challenges facing modern-day healthcare: at least 1.2 million deaths were directly attributed to bacterial AMR worldwide in 2019 [1]. Selection for AMR genes can occur at low concentrations of antibiotics in the environment [2], as well as during chemotherapy in humans and animals. To compound this issue, many AMR genes are easily transferred between different bacterial taxa by horizontal gene transfer, predominantly by plasmid transfer (reviewed in [3]). In this way, previously susceptible pathogens may gain resistance to antibiotics.

Blocking plasmid uptake in key pathogens and environmental bacteria, or removing resident plasmids from these, may provide a means of preventing or decreasing the level of antimicrobial-resistant bacterial infections (reviewed in [4]). One approach of reversing resistance in target bacteria through AMR plasmid removal is the use of CRISPR-Cas9 or related minimal CRISPR systems, typically delivered on an engineered plasmid by conjugation or by means of an engineered bacteriophage. After uptake of the CRISPR delivery tool by recipient cells, the Cas9 nuclease cleaves a DNA sequence defined by its single guide RNA (sgRNA). Depending on the target gene location, this leads to chromosome cleavage and cell death, or plasmid cleavage and resensitization to antibiotics (reviewed in [5,6]).

CRISPR delivery tools have been engineered using various genetic backbones, for instance non-replicating phage plasmids (phagemids) [7,8], expression vectors [9], or synthetic conjugative or mobilizable plasmids [10,11]. These are effective at blocking transfer of resistance genes into specific strains and can remove AMR genes from them. However, in nature, bacteria are commonly embedded in complex microbial communities consisting of many different bacterial strains and species, in which AMR plasmids can be found in multiple, sometimes phylogenetically distant, strains (epidemic plasmids [12]). To target AMR plasmids across different strains or even species, the currently available narrow host-range CRISPR-Cas delivery tools would need to be individually engineered for each target strain. To overcome such drawbacks, a broad host-range delivery vehicle that can be naturally transferred to a range of bacterial species would be highly suitable for application in bacterial communities.

To address this issue, we sought to design a mobile, broad host-range CRISPR-Cas9 expression system that can block AMR gene uptake in multiple species.

We chose the IncP-1ε plasmid pKJK5 [13] as a template for our CRISPR delivery tool. pKJK5 was previously shown to have a particularly broad host range and to spread effectively through microbial communities derived from soil, pig gut microbiomes and wastewater treatment plants. Using either *Escherichia coli*, *Pseudomonas putida*, or *Kluyvera* sp. as donor species, the plasmid was taken up by species belonging to at least 11 different phyla of both Gram-negative and Gram-positive bacteria [14,16]. The gentamicin resistance-encoding cloning vector pHERD30T [17] was chosen as a target plasmid for proof-of-concept experiments, as it can be maintained by *Escherichia* and *Pseudomonas* spp., is compatible with pKJK5, and encodes no stability genes that may interfere with CRISPR-Cas9 targeting.

Here, we engineer pKJK5 to encode *cas9* and *sgRNA*. We show that this engineered CRISPR-Cas9 delivery tool can be used to protect target cells from AMR plasmid uptake, to remove resident AMR plasmids and apply this tool in a range of bacterial species and isolates.

## Results

We engineered the broad host-range plasmid pKJK5 to carry a CRISPR-Cas9 cassette programmed to block uptake of pHERD30T, a plasmid-encoding Gentamicin resistance gene *aacC1*.

### pKJK5::csg construction

**Fig. 1.**
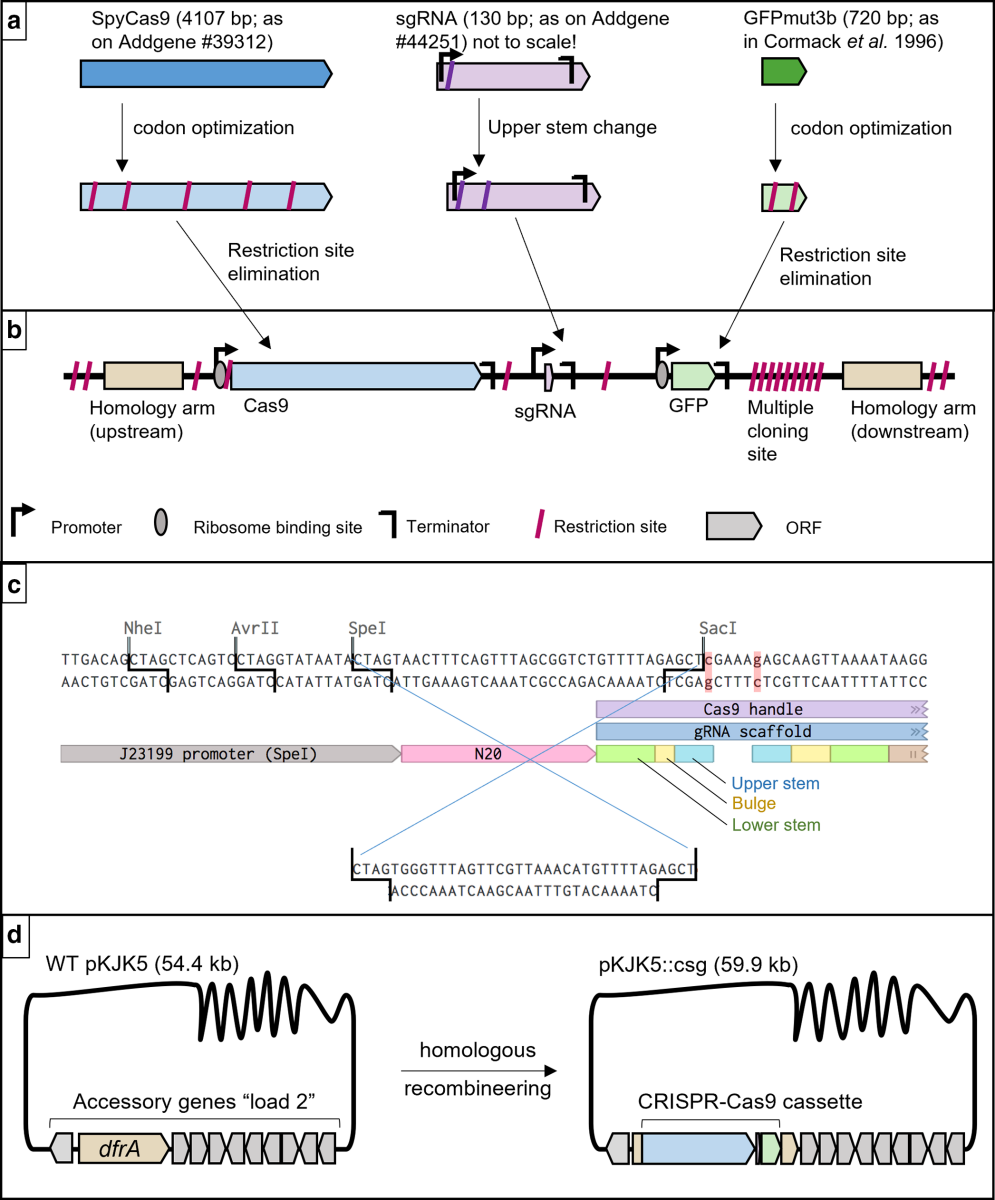
*Insilico* CRISPR-Cas9 cassette construction and pKJK5 recombineering. (a) Source of genes included in cassette (*cas9*, *sgRNA*, *GFPmut3b*) and alterations undertaken. See (c) for *sgRNA* details. Gene length is indicated in base pairs (bp). (b) Final cassette layout. Gene lengths are to scale; spacings, restriction sites, promoters, terminators and ribosome binding sites are not. (c) *sgRNA* region in detail. Highlighted in red: nucleotide mutations introduced in upper stem region to form SacI restriction site. The region to be exchanged for N20 specificity exchange is indicated with blue crossover lines. (d) Homologous recombineering allowed insertion of the CRISPR-Cas9 cassette into *dfrA*, disrupting this gene in pKJK5’s accessory gene load. See the Methods section for details.

First, we designed a CRISPR-Cas9 entry cassette *in silico* that can be recombined with pKJK5. The gene cassette was designed to include the nuclease-encoding gene *cas9*, *sgRNA*, which determines its targeting specificity, and *GFP* (green fluorescent protein) to track plasmid transfer (Fig. 1a). Strategic restriction sites were incorporated in the gene cassette design to ensure full modularity (Fig. 1b) . The sgRNA gene was edited to allow simple exchange of the specificity-defining 20 nt stretch (N20) (Fig. 1c). GFP was added under control of the lacI-repressible promoter P_A1/04/03_ [18] to allow optional repression of GFP expression. The entire CRISPR-Cas9 entry cassette was flanked by homology arms matching the trimethoprim resistance gene *dfrA* on pKJK5 to allow homologous recombination.

This gene cassette was recombined with pKJK5 using homologous λ-red recombineering to yield pKJK5::csg (encoding the genes *cas9, sgRNA, GFP*; see (Fig. 1d) and Methods section). To test the ability of pKJK5::csg to target AMR plasmids, we generated two pKJK5::csg variants with different sgRNA specificities: pKJK5::csg[aacC1] targets gentamicin resistance gene *aacC1* on pHERD30T. As a non-targeting (nt) control, pKJK5::csg[nt] carries a sgRNA with a random nucleotide sequence not present in the study system.

The nucleotide sequence of pKJK5::csg[aacC1], determined by Illumina sequencing, is published on GenBank under accession number OP921802.

### pKJK5::csg acts as a barrier to AMR plasmid acquisition

**Fig. 2.**
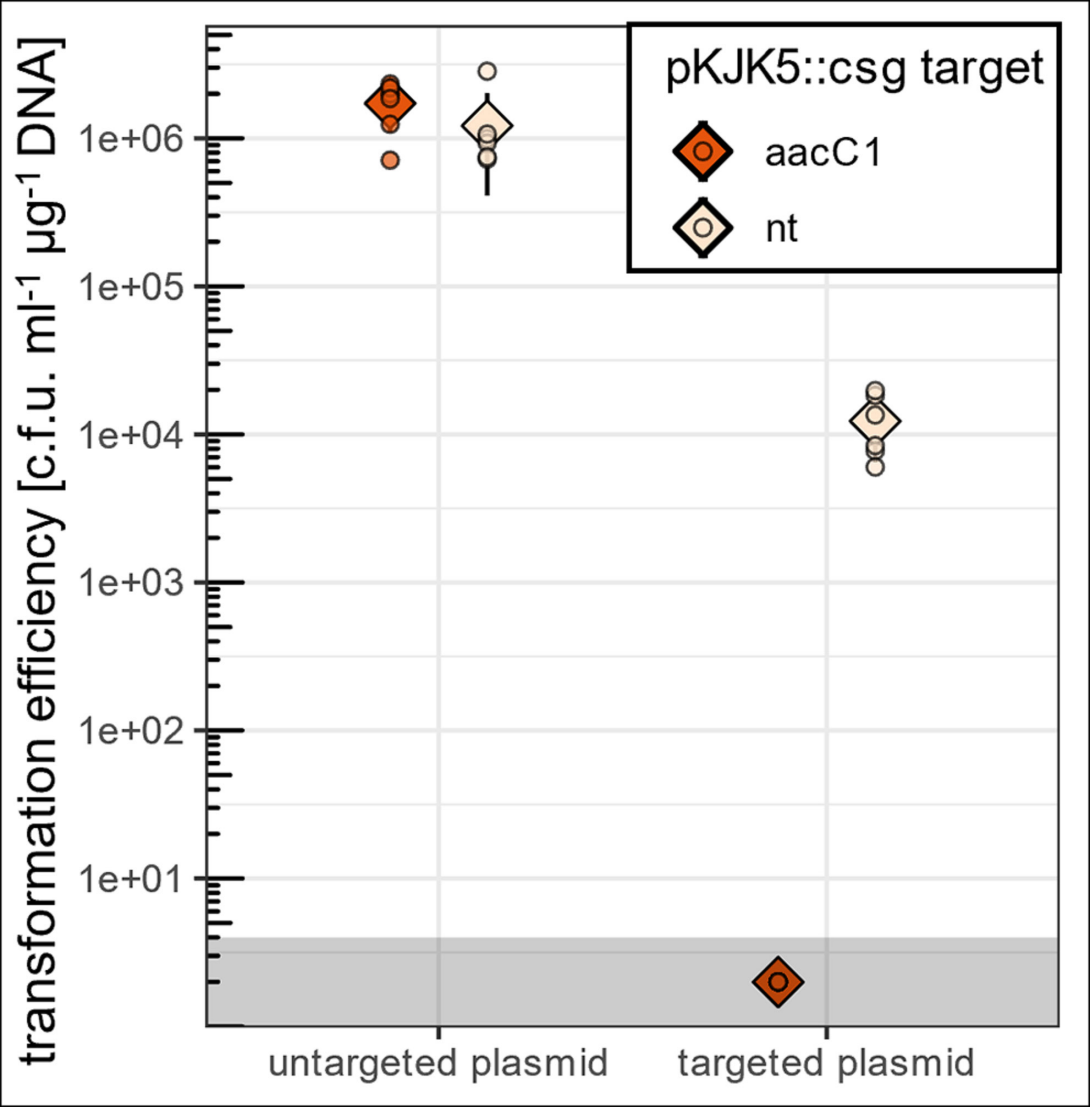
pKJK5::csg as a barrier to plasmid acquisition. Transformation efficiency of *E. coli* DH5α+pKJK5::csg[aacC1]/[nt] with pHERD20T (untargeted plasmid) or pHERD30T (targeted plasmid). [aacC1] transformation with pHERD30T did not yield any transformants, data points are displayed as ½ of the limit of detection. Grey box, data points underneath the limit of detection. *n*, 6, diamonds, mean, circles, individual data points.

To test whether pKJK5::csg can act as a barrier to plasmid acquisition, we measured the transformation efficiency of a targeted plasmid (pHERD30T) or an untargeted control plasmid (pHERD20T) in *E. coli* carrying pKJK5::csg[aacC1]/[nt]. Instead of pHERD30T’s *aacC1* gentamicin resistance gene, pHERD20T encodes ampicillin resistance gene *blaTEM* and is not targeted by either sgRNA. Accordingly, the control plasmid’s transformation efficiency was high regardless of pKJK5’s sgRNA specificity (~10^6^ c.f.u. ml^−1^ µg^−1^ DNA; Fig. 2). In contrast, for the targeted plasmid no successful transformants of DH5α+pKJK5::csg[aacC1] could be recovered. The same plasmid showed transformation efficiencies of ~10^4^ c.f.u. ml^−1^ µg^−1^ DNA in DH5α+pKJK5::csg[nt]. This means that transformation efficiency of a targeted plasmid was reduced to at least the limit of detection (4 c.f.u. ml^−1^ µg^−1^ DNA) in the presence of a targeting CRISPR-Cas system and was nearly four orders of magnitude lower than in the presence of the non-targeting control.

### pKJK5::csg can conjugatively remove resident plasmids

Having established that pKJK5::csg is able to prevent AMR plasmid uptake, we then tested whether it also allowed removal of resident AMR plasmids from target bacterial strains.

To test this, we allowed both versions of pKJK5::csg (targeting and non-targeting) to transfer from an *E. coli* DH5ɑ donor to target bacterium *E. coli* K12 carrying plasmid pHERD30T by mixing overnight recipient and donor cultures in liquid matings.

**Fig. 3.**
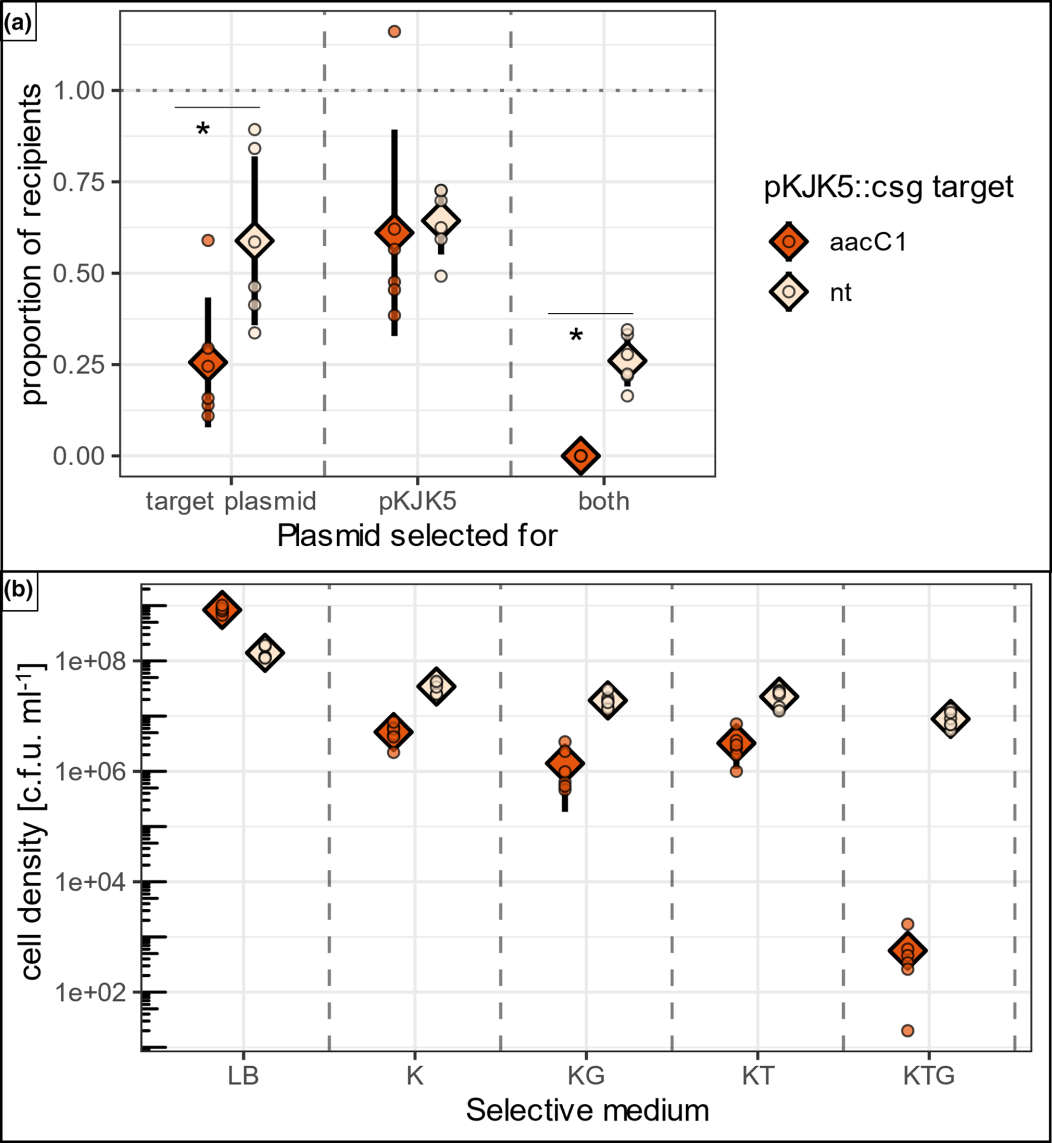
pKJK5::csg can conjugatively remove resident target plasmids. Means (diamonds) and standard deviation (lines) of various colony counts. (a) Proportions of recipients with various plasmid content. Colony counts on plates selecting for recipients+target plasmid, recipients+pKJK5, or recipients+both plasmids divided by colony counts on plates selecting for recipients only, giving proportions of recipients with various plasmid contents; *n*=6. The dotted line indicates 100 %. *These treatments are significantly different as analysed by fitting a binomial GLM followed by Tukey’s post-hoc test, *P*<0.05. (b) Cell density on various selective plates. Colony counts on all different selective plates given in colony-forming units per ml of culture (c.f.u. ml^−1^); *n*=6. LB, LB agar without selection. K, kanamycin, selects for recipients. G, gentamicin, selects for pHERD30T. T, tetracycline, selects for pKJK5::csg. A generalized linear model and Tukey’s post-hoc test revealed that numbers of colonies are different between treatments for all selective media; *P*<0.001; F=273.9; d.f.=11 and 60; R^2^=0.98. See the Methods section for details.

This revealed that most recipients carried pHERD30T after conjugation of the non-targeting pKJK5::csg[nt] control (Fig. 3a; 58.8±23.1% standard deviation from proportional data). In comparison, pKJK5::csg[aacC1] significantly reduced the pHERD30T-carrying proportion by approximately twofold after overnight mating (25.6±17.8 %; *P*=0.015 as assessed by a binomial GLM and Tukey’s post-hoc analysis). This demonstrates that pKJK5::csg can successfully remove a targeted AMR plasmid. pKJK5::csg was taken up by the recipients in equal measures (*P*=1), regardless of Cas9 target (61.0±28.2 % of recipients formed pKJK5::csg[aacC1] transconjugants; 64.4±9.2 % formed pKJK5::csg[nt] transconjugants), indicating that CRISPR-Cas9 targeting did not interfere with pKJK5::csg conjugation. Additionally, selective plating for both the target and the CRISPR plasmid demonstrated that these plasmids tend not to coexist when Cas9 is programmed to target pHERD30T, as only 0.012±0.0097 % of recipients contained both plasmids after conjugation of pKJK5::csg[aacC1]. In contrast, 26±7 % of recipients contained both plasmids after conjugation of the non-targeting control (which is significantly higher; *P*=0.040; Fig. 3a). This is within the margin of error of the value expected from proportions of recipients with other plasmid content (58.8±23.1 % of recipients retained the target plasmid; 64.4±9.2 % took up pKJK5::csg; 0.588×0.644=0.378 or 37.8±20 %).

Raw colony counts revealed that while overall cell densities (‘LB’) were slightly lower for the non-targeting control, the number of recipients (‘K’) was significantly lower when pKJK5::csg[aacC1] was delivered compared with its non-targeting counterpart (Fig. 3b; *P*<0.001; F=273.9, df=11 and 60, R^2^=0.98). This reveals a possible fitness cost of CRISPR-mediated AMR plasmid removal.

Overall, the conjugative delivery of pKJK5::csg using a donor strain led to CRISPR-mediated removal of a targeted plasmid from part of a recipient population.

### pKJK5::csg is a broad host-range barrier to plasmid acquisition

Finally, we tested the ability of pKJK5::csg to act as a barrier to plasmid acquisition in a broader range of bacterial species. To this end, pKJK5::csg carrying transconjugants originating from several environmental, animal and human-associated coliform isolates, as well as of two species of *Pseudomonas* (Table S1), were each transformed with pHERD30T.

**Fig. 4.**
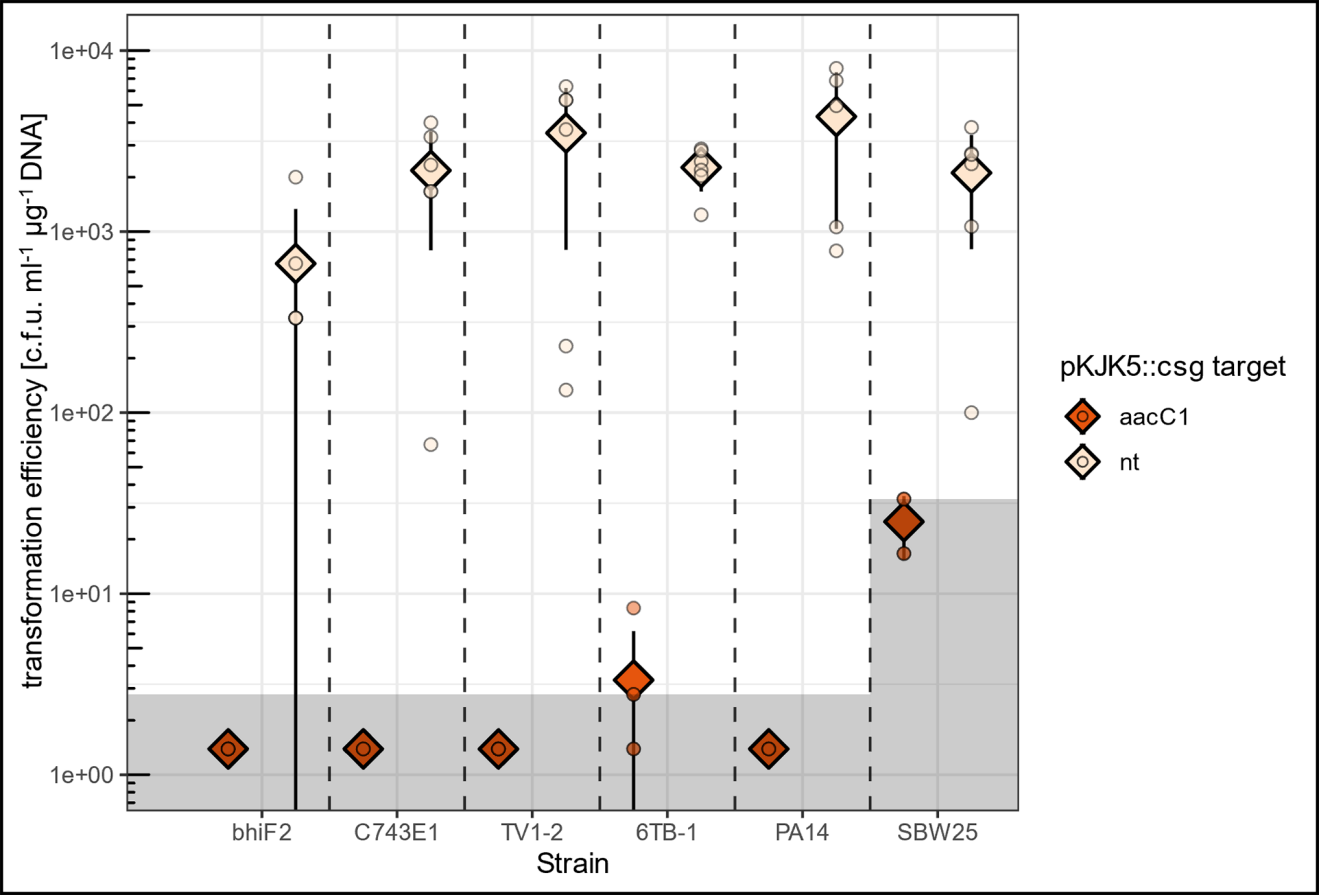
pKJK5::csg prevents transformation of various isolates with a targeted plasmid. Transformation efficiency of various isolates carrying pKJK5::csg[aacC1] or pKJK5::csg[nt] with target plasmid pHERD30T. Diamonds and lines indicate mean±standard deviation, points indicate individual replicates; *n*=3–6. Shaded areas indicate the limit of detection; counts of 0 were manually set to ½ of the limit of detection. bhiF2, C743E1, TV1-2, 6TB-1, coliform pig faeces, environmental and human isolates. PA14, *Pseudomonas aeruginosa* PA14. SBW25, *Pseudomonas fluorescens* SBW25. Transformation efficiency of aacC1 and nt treatments are significantly different for all strains; *P*<0.001 as assessed by Tukey’s HSD after fitting an inverse Gaussian GLM; F=347.6; df=11 and 48; *P*<2.2×10^−16^; adjusted R^2^=0.9858.

Transformation of those isolates carrying pKJK5::csg[nt] was successful (~666–4320 c.f.u. ml^−1^ µg^−1^ DNA; Fig. 4). In contrast, transformation efficiency of all isolates carrying pKJK5::csg[aacC1] was below, or in few individual replicates immediately above the limits of detection. Therefore, transformation efficiency of pHERD30T when carrying pKJK5::csg[aacC1] remained at least two–three orders of magnitude below the transformation efficiency recorded when carrying pKJK5::csg[nt] in all isolates (*P*<0.001; F=347.6; df=11 and 48; adjusted R^2^=0.99).

Additionally, we tested whether pKJK5::csg can be conjugatively delivered to these strains to remove pHERD30T as a resident plasmid. After delivery of pKJK5::csg by liquid mating or filter mating (which is predicted to increase conjugation efficiency [19]) using *E. coli* donors, most isolates did not show a drop in target plasmid maintenance (Fig. S1, available in the online version of this article). The exception to this was isolate TV1-2, in which we observed modest pHERD30T removal after filter mating (Fig. S2; 63.9±32.8 % after non-targeting vs 24.8±19.3 % after targeting treatment; *P*=0.016 as assessed by a binomial GLM and Tukey’s post-hoc analysis). Despite undetectable plasmid removal at a population level, a drop of target plasmid maintenance in all coliform isolates was seen when specifically assessing the transconjugant proportion of the population (Fig. S3; *P*<0.05 for all isolates except those with transconjugant formation close to or below the limit of detection; assessed by Gaussian GLM and Tukey’s post-hoc test).

Overall, pKJK5::csg proved to be an efficient barrier to uptake of a plasmid containing a targeted AMR gene. This was effective in a range of species of laboratory as well as environmental, animal-, and human-associated isolates without the need for re-engineering of pKJK5::csg. While conjugative removal of a resident plasmid from these isolates was undetectable or modest on a population level, target plasmid removal in transconjugants confirmed pKJK5::csg activity after conjugation in this isolate library.

## Discussion

Several previous studies aimed to resensitize bacteria by conjugatively delivering an engineered CRISPR plasmid [10,11, 20,23], but most of these deployed a mobilizable CRISPR delivery tool that requires either a second conjugative plasmid or an engineered donor strain [11,20, 22,24]. Crucially, the application of a CRISPR delivery tool has been shown to be more effective when conjugative machinery and CRISPR machinery are encoded on the same genetic element (in *cis*), rather than on separate plasmids (in *trans*) [25]. Therefore, we generated the fully conjugative plasmid pKJK5::csg, and demonstrated that it protects a range of host species from uptake of targeted AMR plasmid pHERD30T. Additionally, conjugation of pKJK5::csg led to removal of pHERD30T from a recipient strain.

CRISPR delivery tools could provide a means of tackling hotspots of horizontal gene transfer and reservoirs of AMR genes by removal of AMR plasmids: the human gut microbiome [26] and environments such as livestock farms or wastewater [27] see frequent exchange of resistance genes between different bacterial species, including pathogens. Thanks to the broad natural host range of the plasmid it is derived from [14,16], pKJK5::csg is particularly promising for the application in such microbially diverse environments and could help to either remove AMR plasmids from them (as in Fig. 3), or to protect microbiomes from becoming colonized by AMR plasmids (as in Fig. 4). All genetic cargo of pKJK5::csg (*cas9*, *sgRNA*, *GFP*) was inserted into trimethoprim resistance gene *dfrA*, which sits within the accessory gene pool of pKJK5 [13]. Therefore, we can expect pKJK5’s broad transfer range into at least 11 phyla to be maintained for pKJK5::csg.

In previous work, AMR plasmid removal was limited by the low conjugation efficiency of the CRISPR plasmid. In our work, conjugation efficiency was also modest: conjugation of pKJK5::csg to recipients failed in almost half of the cases (only ~61–65 % of recipients formed transconjugants; Fig. 3a). In contrast, once pKJK5::csg was present in target cells, CRISPR targeting only failed in ~1 in 5000 cases (~0.02 % of pKJK5::csg[aacC1] transconjugants also contained pHERD30T; Table S2). Furthermore, conjugation efficiency for the different isolates (Fig. S4) varied depending on recipient identity. As pKJK5::csg was active in all cases once present in recipients (Fig. S3), we suggest that target plasmid removal using pKJK5::csg could primarily be improved by optimizing this CRISPR delivery tool’s conjugation efficiency. For instance, identification of a suitable donor for a target community is paramount: conjugation of wild-type pKJK5 to a soil community was more effective using *E. coli* as a donor than when using *Kluyvera* sp. or *Pseudomonas putida* [14]. Furthermore, plasmids can evolve higher transfer rates as a trade-off against increased plasmid cost – as observed for R1 when cultured under conditions with ample naïve hosts [28]. Perhaps directed evolution could achieve the same for pKJK5::csg.

Although conjugation efficiency is likely to be a highly important factor for optimization, CRISPR-mediated target plasmid removal outcome may also depend on other variables. For example, in our work pKJK5::csg[aacC1] reduced the efficiency of target plasmid transformation compared with pKJK5::csg[nt] to different extents in different species (Fig. 4), which may be related to variation in pKJK5::csg fitness costs and maintenance. We previously observed this phenomenon for a closely related plasmid, pKJK5::*gfp*^PL^, the cost of which was associated with plasmid maintenance and varied between different hosts and growth contexts [29]. This plasmid cost is likely due to constitutive costs of Cas9 and sgRNA expression, which can have different extents in different species [30,32].

Beyond this, target plasmid removal efficiency by CRISPR delivery tools could depend on other factors, such as target plasmid mobility [11], plasmid copy number [33], or the presence of other payload genes on target plasmids or in target genomes such as anti-CRISPR proteins [34] or toxin–antitoxin systems. These should be further experimentally investigated for optimization of AMR plasmid removal using CRISPR-Cas9.

In summary, CRISPR delivery tools which target and cleave AMR plasmids may be used to protect their bacterial hosts from plasmid uptake or to resensitize them to antibiotics by removing resident plasmids. Proof-of-concept experiments have achieved this in simple set-ups, but in nature bacteria are embedded in complex communities so a broad host-range CRISPR delivery tool is needed. This work establishes pKJK5::csg as a broad host-range CRISPR delivery tool with the ability to remove AMR plasmids from diverse bacterial species, and thereby forms a basis for interventions aimed at clearing AMR genes from bacterial communities.

## Methods

### Strains, growth conditions and molecular cloning

Bacterial strains, plasmids and primers are listed in Table 1. Unless otherwise specified, all strains were cultured in LB at 37 °C with shaking at 180 r.p.m. Where necessary for plasmid selection, antibiotics were added at the following concentrations: Ap – 100 µg ml^−1^ ampicillin; Carb – 250 µg ml^−1^ carbenicillin; Cm – 25 µg ml^−1^ chloramphenicol; Gm – 50 µg ml^−1^ gentamicin; Km – 50 µg ml^−1^ kanamycin; Sm – 50 µg ml^−1^ streptomycin; Tc – 12 µg ml^−1^ tetracycline; Tmp – 10 µg ml^−1^ trimethoprim. Where necessary, the following additives were added to growth media after preparation of stock solutions and filter-sterilization: Ara – 0.5 % (w/v) arabinose; Gluc – 0.2 % (w/v) glucose.

**Table 1.** Strains, plasmids and primers

***Strains***			
**Name**	**Shorthand**	**Notes**	**Reference**
*E. coli* DH5α	DH5α		Common laboratory strain
*E. coli* K12::mCherry	K12::mCherry	Chromosomal Km^R^, mCherry, lacI insertion	[14]
*E. coli* MFDpir	MFDpir	Auxotrophic, needs DAP	[42]
*Escherichia/Shigella* pig gut isolate bhiF2	bhiF2	Pig faeces isolate	This study
Coliform isolate C743E1	C743E1	Human rectal swab isolate	[43]
Coliform isolate TV1-2	TV1-2	Sewage water isolate	[43]
Coliform isolate 6TB-1	6TB-1	Bathing water isolate	[43]
*P. aeruginosa* PA14	PA14	Burn patient clinical isolate	[44]
*P. fluorescens* SBW25	SBW25	Sugar beet isolate	[45]
***Plasmids***			
**Plasmid**	**Resistance and payload**	**Notes**	**Reference**
pMARQ_csg [aacC1]/[nt]	Ampicillin; CRISPR-Cas9 cassette		This study
pHERD20T	Ampicillin		[17]
pHERD30T	Gentamicin		[17]
pDOC-K	Ampicillin; kanamycin		[41]
pDOC	Ampicillin	Km^R^ deleted from pDOC	This study
pDOC_csg[aacC1]/[nt]	Ampicillin	Recombineering template	This study
pACBSCE	Chloramphenicol, Sce-I, λ-red		[41]
pKJK5	Tetracycline, trimethoprim		[13]
pKJK5::csg[aacC1]	Tetracycline, CRISPR-Cas9 cassette	GenBank OP921802	This study
pKJK5::csg[nt]	Tetracycline, CRISPR-Cas9 cassette	Non-targeting control	This study
***Primers and sequences***			
**Name**	**Sequence (5’ → 3’**)		
[aacC1]	AAGTTAGGTGGCTCAAGTAT		
[nt]	GGTAAGACCATTAGAAGTAG		
N20_aacC1_top	CTAGTAAGTTAGGTGGCTCAAGTATGTTTTAGAGCT		
N20_aacC1_btm	CTAAAACATACTTGAGCCACCTAACTTA		
N20_nt_top	CTAGTGGTAAGACCATTAGAAGTAGGTTTTAGAGCT		
N20_nt_btm	CTAAAACCTACTTCTAATGGTCTTACCA		
dfrA_fw	GTGAAACTATCACTAATGGTAG		
dfrA_rv	TTAACCCTTTTGCCAGATTT		
Cas9_bw	ATGCTGTACTTCTTGTCCAT		
GFPend_fw	CATGGACGAACTGTATAAGT		

Where *E. coli* MFDpir was used, cultures were supplemented with 300 mM DAP (diaminopimelic acid) to ensure growth of this auxotrophic strain. By omitting DAP, the strain could be selected against.

Pig faeces isolate bhiF2 was isolated from a microbial pig gut community. Briefly, pig faecal samples, collected from four Cornish black pigs, were suspended in 10 % glycerol and 0.9 % (w/v) NaCl, and subsequently blended and strained. The resulting pig faeces slurry was plated onto BHI (brain heart infusion) agar plates without selection, and bhiF2 was one of several visually distinct bacterial isolates picked from these plates. Genus identity was confirmed as *Escherichia/Shigella* by 16S colony PCR, Sanger sequencing and blast homology search.

All molecular cloning steps were carried out with high-fidelity restriction enzymes (NEB) and according to manufacturer protocols, using commercially chemically competent *E. coli* DH5α cells (NEB).

#### *In silico* cassette construction and specificity swap

The CRISPR-Cas9 gene cassette was constructed and restriction sites were identified using Benchling [35]; an overview of the workflow is shown in Fig. 1. Sources of nucleotide sequences for each module are summarized in Table 2.

**Table 2.** Sequence sources of CRISPR-Cas9 cassette coding and non-coding elements

Element	Source
Cas9	Addgene plasmid #39 312 [46]. Coding sequence only
sgRNA	Addgene plasmid #44 251 [39]; N20 replaced to target *aacC1*. Constitutive promoter and terminator as in source. Upper stem edited as described below
GFPmut3b	[47]
Multiple cloning site	pBAM1 [48]. The final version is heavily edited to exclude restriction sites used elsewhere
Cas9 promoter/terminator	Constitutive promoter as found on pBAM1 [48]: *bla* Ampicillin resistance upstream region (70 nts) with two final nucleotides changed to CC (to create NcoI restriction site for promoter exchange), downstream region (54 nts) as terminator
GFP promoter	P_A1/04/03_ as found on GenBank acc. no. DQ493878. Constitutive, LacI-repressible promoter with strong ribosome binding site
GFP terminator	*neo* Kanamycin resistance downstream region (29 nts) as found on pBAM1 [48]
Homology arms	Upper homology: nt 450–550; lower homology: nt 551–651 of *dfrA* on pKJK5 (GenBank accession AM261282.1)

Genes were codon-optimized using OPTIMIZER [36] with pKJK5 codon usage database tables [37]. Common restriction sites were removed from these coding sequences by changing codons to the second most common on pKJK5. When creating or altering multiple cloning sites, random nucleotides were added to increase spacing and allow double digestions. Terminator presence (and absence from unwanted regions) was checked using Arnold [38].

The single guide RNA (sgRNA) gene was placed under the control of constitutive promoter J23119 (which contains a SpeI restriction site; as on pgRNA [39]) and was edited to encode a SacI restriction site in its upper stem region, the function of which is generally resilient to mutations [40]. These two restriction sites allow simple exchange of the specificity-defining N20 stretch on the sgRNA.

The CRISPR-Cas9 gene cassette was commercially synthesized (Thermo Scientific) and delivered on vector pMA-RQ_csg. To exchange sgRNA target specificity of pMARQ_csg, DNA oligonucleotides containing a 20 nt specificity region with SpeI- and SacI-compatible overhangs (Table 1; N20_aacC1_top/btm; N20_nt_top/btm) were annealed by mixing 10 µl of each 100 µM oligo with 80 µl of annealing buffer (100 mM potassium acetate, 30 mM HEPES, pH=7.5) and heating to 95 °C followed by slow overnight cooling to room temperature. Subsequently, the annealed oligos were phosphorylated using T4 polynucleotide kinase (NEB) according to the manufacturer’s instructions. The annealed and phosphorylated oligos were inserted between pMARQ_csg’s SpeI and SacI restriction sites following standard molecular cloning protocols, resulting in pMARQ_csg[aacC1] and pMARQ_csg[nt] (Fig. 1c).

### pKJK5::csg recombineering

The CRISPR-Cas9 cassette was introduced to pKJK5 using homologous recombineering with an altered version of pDOC-K and pACBSCE plasmids [41]. The following steps were carried out in parallel with pMARQ_csg[aacC1] and pMARQ_csg[nt]. To construct pDOC_csg as a template vector containing the CRISPR cassette, the kanamycin resistance gene was removed from pDOC-K using AvrII and NheI restriction sites, gel extraction (Qiagen gel extraction kit) and religation of the 5.9 kb band. Next, the CRISPR-Cas9 cassette was inserted from pMARQ_csg using restriction sites EcoRI and HindIII to create pDOC_csg.

*E. coli* DH5α+pKJK5 was transformed with pACBSCE and pDOC_csg following standard procedures for electrotransformation of *E. coli*. Briefly, log-phase *E. coli* were washed twice with ice-cold 10 % (w/v) glycerol and concentrated ~30 times. Cells were electroporated by applying 1.8kV in 2 mm gap cuvettes.

Cells were cultured in the presence of Tc+Tmp (pKJK5)+Ap (pDOC_csg)+Cm (pACBSCE) to maintain plasmids, and in the presence of Gluc to prevent leaky λ-red expression.

Then 10 µl of an overnight culture of this recombineering-ready strain were used to inoculate 1 ml LB+Tc+Tmp+Cm+Ap+Gluc at 37 °C and grown at 250 r.p.m. for 2 h in triplicate. The cultures were spun and resuspended in 1 ml LB+Tc+Ara and incubated at 37 °C until turbid (4–5 h) to allow recombination. Finally, the cultures were plated onto LB +Tc+5 % sucrose in several dilutions and incubated at 28 °C for 48 h (to allow counterselection of bacteria with intact pDOC_csg plasmids). To isolate recombinants, bacterial lawns were investigated for GFP expression using a fluorescence compound microscope (Olympus BX61 with U-MWIB3 mirror turret). Green colonies were restreaked onto LB+Tc several times until all colonies appeared GFP+, indicating successful recombination events. Next, GFP+ colonies were checked for correct CRISPR cassette insertion by colony PCR using primer combinations dfrA_fw / Cas9_bw and GFPend_fw / dfrA_rw (Table 1), one primer of which annealed to the pKJK5 backbone and to the CRISPR-Cas9 cassette, respectively.

### Illumina sequencing

pKJK5::csg[aacC1] plasmid DNA was extracted using a Thermo Fisher Midiprep kit. Plasmid DNA was Illumina-sequenced by the University of Liverpool Centre for Genomic Research. Bioinformatics analyses and sequence assembly were carried out using Bash and Conda.

We analysed read quality using FastQC v0.11.9 and discarded poor-quality reads using Trimmomatic v0.39. Assembly was carried out using SPAdes v3.15.2 with the settings --plasmid and --careful. QUAST v5.0.2 was used to try multiple settings and select the best assembly. Finally, SPAdes-generated contigs were visualized using Bandage v0.8.1 and aligned to GenBank-deposited pKJK5 (AM261282.1) and *E. coli* K12 sequences (NZ_CP010444.1). Contigs that aligned to *E. coli* K12 were discarded, which yielded a single circular contig encoding pKJK5 and the CRISPR-Cas9 cassette. This contig contained a 127 bp duplication as an artefact of circularization, which we removed as a post-processing step. pKJK5::csg[aacC1] is identical to its theoretical sequence, except for a single-nucleotide deletion in the pKJK5 backbone 12 nt upstream of *trfA*. pKJK5::csg[aacC1] is deposited on GenBank under the accession number OP921802.

### Blocking plasmid uptake in *E. coli*

Electrocompetent *E. coli* strains were prepared using standard protocols as described above.

*E. coli* DH5α+pKJK5::csg[aacC1]/[nt] were electroporated with 500 ng of plasmid DNA (pHERD30T/pHERD20T) in six replicates. Fifty microlitres of transformed cells were plated onto selective plates (LB+Gm/Amp) and transformation efficiency was calculated for each strain (c.f.u. ml^−1^ µg^−1^ DNA). Where no colonies could be recovered, transformation efficiency was set to ½ of the limit of detection (transformation efficiency if a single colony was recovered). Plating transformation mixes onto double selective plates (LB+Tc+ Gm/Amp) yielded a similar amount of colonies (not shown).

### *E. coli* conjugation experiments

For liquid mating, single colonies of donors (*E. coli* DH5α+pKJK5::csg[aacC1]/[nt]) and recipients (*E. coli* K12::mCherry+pHERD30T) were suspended and grown overnight in 5 ml each LB+Tc or LB+Gm, respectively. Cultures were washed twice with 0.9 % (w/v) NaCl and 50 µl of donors and recipients were co-incubated in fresh 5 ml LB microcosms in six replicates and incubated overnight at 37 °C, 50 r.p.m. The next day, all cultures were frozen in 20 % (w/v) glycerol at −70 °C and plated onto various selective media: LB without selection allows donors and recipients to grow, LB+Km selected for all recipients, LB+Km+Tc selected for recipients that had taken up pKJK5::csg (transconjugants), LB+Km+Gm selected for recipients with target plasmid pHERD30T, and LB+Km+Gm+Tc selected for recipients containing both plasmids. All selective plates were also analysed for GFP expression as above, and GFP expression was found to be as expected (GFP+ colonies when pKJK5::csg was selected for). Additional controls (not shown) included donor-only and recipient-only controls, and yielded colonies as expected. Enumerating colonies on selective plates allowed us to calculate the proportions of recipients carrying various plasmids.

### Broad host-range barrier to plasmid uptake

Further information on isolates used in this experiment is given in Table S1.

Using *E. coli* DH5α or *E. coli* MFDpir as a donor, pKJK5::csg[aacC1]/[nt] transconjugants of bhiF2, C743E1, TV1-2, 6TB-1, *Pseudomonas aeruginosa* PA14, and of *Pseudomonas fluorescens* SBW25 were generated and pKJK5::csg[nt]/[aacC1] was selected for and maintained with Tc (except for SBW25, where selection at this Tc concentration failed and transconjugants were selected by identifying GFP+colonies). Next, each strain was made electrocompetent and transformed with 600 ng pHERD30T in six replicates following the *E. coli* electroporation protocols described above (bhiF2, C743E1, TV1-2, 6TB-1). *P. aeruginosa* PA14 was made electrocompetent by washing 1 ml aliquots of an overnight culture twice with a 300 mM sucrose solution followed by resuspension in 100 µl 300 mM sucrose. To prepare electrocompetent *P. fluorescens* cells, the protocol for PA14 was followed with the exception that SBW25 was grown at 28 °C, cultures were grown until log phase in the absence of Tc selection (estimated OD_600_ : 0.5–0.6) and then the protocol was started. *Pseudomonas* cultures were electroporated at 2.5kV in 2 mm gap cuvettes in six replicates, and recovered in 1 ml SOB at 37/28 °C, 250 r.p.m. for 1 h. Samples that arced during electroporation were discarded, yielding *n*=3–6 for all samples.

Eight hundred microlitres of all strains were plated onto LB+Gm and transformation efficiency calculated as described above. For *P. fluorescens* SBW25 transformations, only 50 µl of transformed cells were plated, resulting in a higher limit of detection.

As a control for competence, one replicate of each competent strain carrying pKJK5::csg[aacC1] was transformed with pHERD30T_mut, a pHERD30T-derivative that encodes a mutated *aacC1* gene and is therefore not targetable by pKJK5::csg. All controls yielded colonies, indicating successful transformation and competence of strains.

#### Conjugative removal from diverse isolates

In order to assess whether pKJK5::csg could be used to conjugatively remove pHERD30T from this library of isolates, we transformed each of the isolates with pHERD30T after preparing electrocompetent cells as described above.

We suspended individual colonies of donors (*E. coli* DH5α+pKJK5::csg[aacC1]/[nt]) and of recipients (bhiF2+pHERD30T, C743E1+pHERD30T, TV1−2+pHERD30T, 6TB-1+pHERD30T, PA14+pHERD30T, SBW25+pHERD30T) in LB containing appropriate antibiotics for plasmid maintenance (Tc/Gm). After overnight incubation at 37 °C (28 °C for SBW25), cultures were washed twice with 0.9 % NaCl and resuspended to OD_600_=0.6.

In parallel, we carried out liquid mating as described above for *E. coli* and solid-surface filter mating, in a donor : recipient ratio of 100 : 1. In brief, 1 ml each of OD-adjusted donor cultures and of 1 : 100 diluted recipient cultures were concentrated onto a 0.2 µM pore size cyclopore membrane by applying a vacuum, and membranes were placed onto 10 % LB plates and incubated at 37 °C (28 °C for SBW25) overnight (*n*=5). Membranes were placed into 3 ml 0.9 % NaCl and vortexed to recover cells. In addition to the usual donor-only and recipient-only controls, sterility controls were carried out to check for sterility of the vacuum pump and filter system, and no colonies were recovered on any selective medium.

Cell suspensions of recovered cells after filter mating as well as after liquid mating were plated onto various selective media in 5 µl droplets from a dilution series ranging from 10^0^ (undiluted) to 10^−7^. Cultures were plated onto LB (all donors and recipients), LB+Ap (all recipients), LB+Ap+Tc (transconjugants), LB+Ap+ Gm (recipients with target plasmid) and LB+Ap+Tc+Gm (recipients with both plasmids). For PA14 samples, Ap was replaced with Km. For SBW25 samples, Tc was replaced with 120 µg ml^−1^ tetracycline. PA14 recipient-only controls showed a low amount of small colonies on plates containing Tc, so these plates were not used in any analyses for treatments containing PA14.

### Statistical analyses

Data processing, data visualization and statistical analyses were carried out using R software version 4.1.0 and RStudio version 1.4.1717 with the following packages: tidyverse version 1.3.1, janitor version 2.1.0, arm version 1.11–2, MuMIn version 1.43.17, bbmle version 1.0.24, ggpubr version 0.4.0, lemon version 0.4.5, purrr version 0.3.4, lubridate version 1.7.10, lme4 version 1.1–27.1 and LMERConvenienceFunctions version 3.0

Generalized linear models (GLMs) were fitted to data as listed below using the base R ‘glm’ function; additional details are given below and in Table S3. For all models, homogeneous variance of residuals, normal distribution of residuals, explanatory variable collinearity and absence of bias by influential observations were tested by plotting data and using the plot(model) function and these assumptions were found to be upheld. Other model types, link functions and model structures were tested and the models that satisfied assumptions the best during model validation were chosen. Inclusion of other explanatory variables was tested, and non-significant variables were dropped. Where mentioned, a Tukey’s post-hoc test was carried out to assess statistical difference between treatment categories.

#### pKJK5::csg conjugative delivery: target plasmid retention (Fig. 3a)

First, numerical proportions of >1 were set to 1 to allow for proportional data analysis (one datapoint for the pKJK5::csg[aacC1] treatment when selecting for pKJK5 only). Next, we fitted a binomial GLM with logit link function describing proportion as a function of pKJK5::csg target, selective medium and their interaction. Raw colony counts on plates selecting for recipients (K) were used as weights. The binomial model structure was chosen to suit the proportional data.

F=0.397; df=7 and 40; pseudo R^2^=0.868.

A Tukey’s post-hoc test revealed statistical likelihood of differences between treatments for each selective medium:

TK (transconjugants) *P*=0.995; KG (target plasmid) *P*=0.0149; TKG (both plasmids) *P*=0.0406.

#### pKJK5::csg conjugative delivery: cell densities (Fig. 3b)

A Gaussian GLM was fitted with an identity link function; explaining log-transformed colony counts in c.f.u. ml^−1^ with pKJK5::csg target, selective plate and their interaction. Tukey’s post-hoc honest significance differences were carried out to assess the difference between colonies on recipient-selecting plates for the targeting treatment and the non-targeting control (*P*=0.000017). This difference between treatments was significant for all selective media (*P*<0.001). Gaussian model structure was chosen to suit the approximately normally distributed log-transformed data.

F=273.9; d.f.=11 and 60; R^2^=0.98.

#### pKJK5::csg prevents transformation in various host backgrounds (Fig. 4)

An inverse Gaussian GLM was fitted with log link function describing log(transformation efficiency) as a function of host strain, pKJK5::csg target and their interaction. The inverse Gaussian model structure was chosen to suit the zero-bounded data.

F=374.6; d.f.=11 and 48; *P*<2.2×10^−16^; adjusted R^2^=0.9858.

#### Conjugative pKJK5::csg delivery to diverse isolates (Fig. S1)

An inverse Gaussian GLM was fitted with log link function describing target plasmid proportion as a function of isolate identity, pKJK5::csg target and their interaction. Data from filter mating and from liquid mating were modelled separately. The inverse Gaussian model structure was chosen to suit the zero-bounded data; binomial modelling was unavailable for this full dataset due to high variation and proportions exceeding 1.

Filter mating: F=2.067; d.f.=11 and 48; *P*=0.0419; adjusted R^2^=0.166.

Liquid mating: F=3.682; d.f.=11 and 60; *P*=0.0005; adjusted R^2^=0.2935.

Model coefficients showed significant effects in C743E1 during filter (*P*=0.002) and liquid (*P*=1.5×10^−8^) mating, and for TV1-2 during filter mating (*P*=0.006). TV1-2 was chosen for further analysis due to likely biological relevance (see below); C743E1’s effects are likely attributed to low pHERD30T maintenance regardless of pKJK5::csg treatment.

#### pKJK5::csg conjugative delivery to TV1-2 by filter mating (Fig. S2)

We fitted a binomial GLM with logit link function describing proportion as a function of pKJK5::csg target, selective medium and their interaction. A single high-influence data point was removed for analyses (100 % pKJK5 conjugation efficiency in a single targeting replicate). Raw colony counts on plates selecting for recipients (A) were used as weights. The binomial model structure was chosen to suit the proportional data.

F=1.164; df=5 and 23; *P*=0.3567; pseudo R^2^=0.707.

A Tukey’s post-hoc test revealed statistical likelihood of differences between treatments for each selective medium:

AT (transconjugants) *P*=0.505; AG (target plasmid) *P*=0.0157; ATG (both plasmids) *P*=0.187.

#### pHERD30T removal in transconjugant sub-population (Fig. S3)

All data visualizations and analyses were carried out after setting the proportions below to ½ of the limit of detection (the proportion of transconjugants carrying pHERD30T if a single colony was found on the appropriate selective plates). Analyses of PA14 and of SBW25 samples were excluded due to inaccurate assessment of transconjugants (PA14 recipient-only controls grew in the presence of Tc) and a lack of conjugation, respectively.

We fitted a Gaussian GLM with identity link function describing log-transformed proportion as a function of pKJK5::csg target, recipient identity and their interaction. Data from filter and from liquid mating were modelled separately.

Filter: F=59.42; d.f.=7 and 25; *P*=5.27×10^−14^; adjusted R^2^=0.927.

Liquid: F=101.2; d.f.=7 and 40; *P*<2.2×10^−16^; adjusted R^2^=0.933.

A Tukey’s post-hoc test revealed statistical likelihood of differences between treatments for each isolate.

Filter: bhiF2 *P*=0.00017; C743E1 *P*=0.214; TV1-2 *P*=0; 6TB-1 *P*=0.114.

Liquid: bhiF2 *P*=0.00961; C743E1 *P*=0.0460; TV1-2 *P*=0; 6TB-1 *P*=0.0000011.

## supplementary material

10.1099/mic.0.001334 Uncited Supplementary Material 1.

10.1099/mic.0.001334 Uncited Supplementary Data Sheet 1.

## Abbreviations

AMR: antimicrobial resistance
Cas: CRISPR-associated
CRISPR: clustered regularly interspaced short palindromic repeats
GFP: green fluorescent protein
GLM: generalized linear model
sgRNA: single guide RNA
