# Supplementary material for: Removal of AMR plasmids using a mobile, broad host-range CRISPR-Cas9 delivery tool
Source: Microbiology (Reading). 2023 May 25;169(5):001334. doi: 10.1099/mic.0.001334 (PMC10268836; doi:10.1099/mic.0.001334)
Supplement: Uncited Supplementary Material 1. [file mic-169-01334-s001.pdf]

Removal of AMR plasmids using a mobile, broad host-range CRISPR-Cas9 delivery tool.

David Walker-Sünderhauf[1]*, Uli Klümper[2], Elizabeth Pursey[1], Edze R Westra[1], William H Gaze[3], Stineke van Houte[1]*.

1. College of Life and Environmental Sciences, University of Exeter, Environment and Sustainability Institute, Penryn, UK, TR10 9FE

2. Department Hydrosciences, Technische Universität Dresden, Institute of Hydrobiology, Dresden, Germany

3. European Centre for Environment and Human Health, University of Exeter Medical School, Environment and Sustainability Institute, Penryn, UK, TR10 9FE

*corresponding authors; D.Walker-Sunderhauf@exeter.ac.uk; c.van-houte@exeter.ac.uk

Supporting Information

Supplementary Data: All raw data are given in the supplementary .xslx file.

Figure S1: Target plasmid proportions after conjugative delivery of pKJK5::csg to various isolates.

Means (diamonds) and standard deviation (lines) of recipients carrying target plasmid pHERD30T divided by total recipients after delivery of pKJK5::csg to various isolates by solid-surface conjugative filter mating or by liquid mating using E. coli DH5α donors. N=5-6. The dotted line indicates 100%.

bhiF2, C743E1, TV1-2, 6TB-1: coliform pig faeces, environmental, and human isolates. PA14: Pseudomonas aeruginosa PA14. SBW25: Pseudomonas fluorescens SBW25.

When fitting an inverse Gaussian GLM, model coefficients showed significant effects in C743E1 during filter (p=0.002) and liquid ($p=1.5x10^{-8}$) mating, and for TV1-2 during filter mating (p=0.006). See methods for model details.

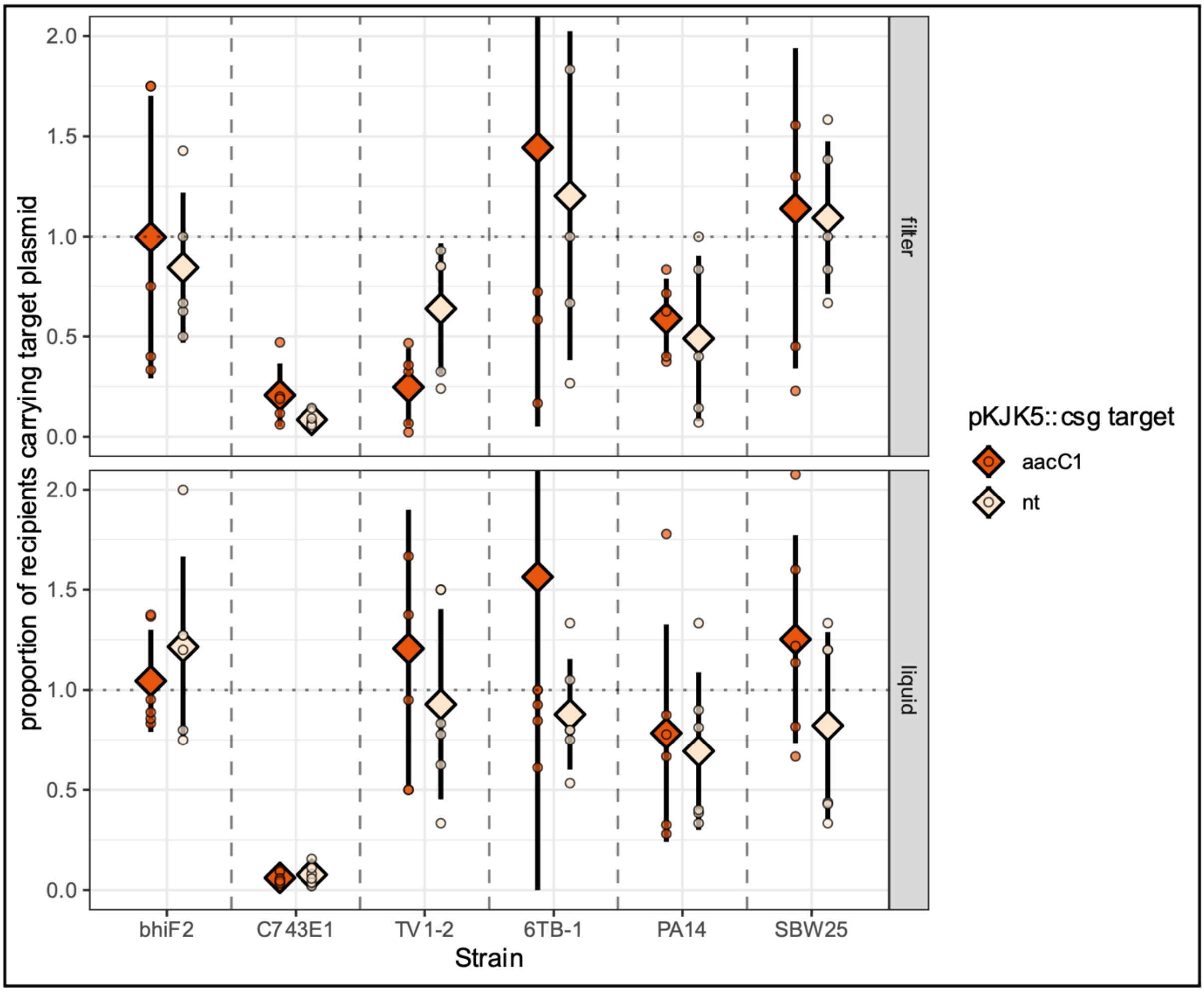
proportion of recipients carrying target plasmid
2.0
1.5
1.0
0.5
0.0
filter
liquid
bhiF2
C743E1
TV1-2
6TB-1
PA14
SBW25
Strain
pKJK5::csg target
aacC1
nt

Figure S2: TV1-2 recipients after pKJK5::csg delivery by filter mating.

Means (diamonds) and standard deviation (lines) of various colony counts after delivery of pKJK5::csg to coliform isolate TV1-2 by solid-surface conjugative filter mating using *E. coli* DH5α donors. These data are from the same dataset as presented in Figure S1; N=5.

Colony counts on plates selecting for recipients+target plasmid, recipients+pKJK5, or recipients+both plasmids divided by colony counts on plates selecting for recipients only, giving proportions of recipients with various plasmid contents; N=5. The dotted line indicates 100%. *these treatments are significantly different as analysed by fitting a binomial GLM followed by Tukey's post-hoc test, p=0.016. F=1.164, d.f.=5&23, pseudo R^2=0.7.

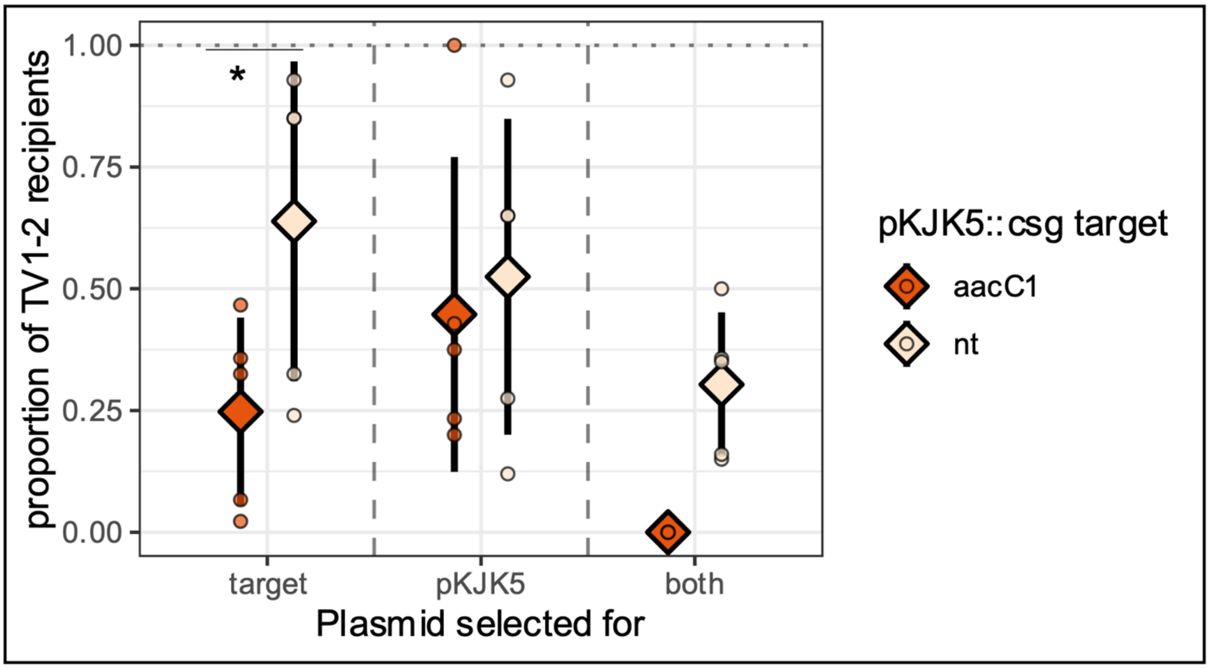

Figure S3: Target plasmid proportions within transconjugants after conjugative delivery of pKJK5::csg to various isolates.

Means (diamonds) and standard deviation (lines) of recipients carrying target plasmid pHERD30T and pKJK5 divided by total recipients carrying pKJK5::csg after delivery of pKJK5::csg to various isolates by solid-surface conjugative filter mating or by liquid mating using *E. coli* DH5α donors. The dotted line indicates 100%. This metric describes target plasmid removal within the fraction of recipients which successfully took up pKJK5::csg. These data are from the same dataset as presented in Figure S1.

Black datapoints indicate those underneath the limit of detection which were manually set to ½ of the limit of detection (the proportion of transconjugants carrying pHERD30T if a single colony was found on the appropriate selective plates). Therefore, black datapoints should be interpreted as a maximum possible value for the corresponding sample. The mean ± standard deviation opacity scales with certainty (i.e. certainty = 1 if all datapoints lie above the limit of detection, and certainty = 0 if no datapoint lies above the limit of detection).

Samples without recovered transconjugants were excluded, resulting in no data for SBW25 and N=2-6 for the remaining treatments. PA14 was excluded from analysis due to growth of recipients without pKJK5::csg on appropriate selective plates.

*$p<0.05$/**$p<0.01$/***$p<0.001$ these treatments are significantly different as analysed by fitting gaussian GLMs followed by Tukey's post-hoc test. See methods for model details.

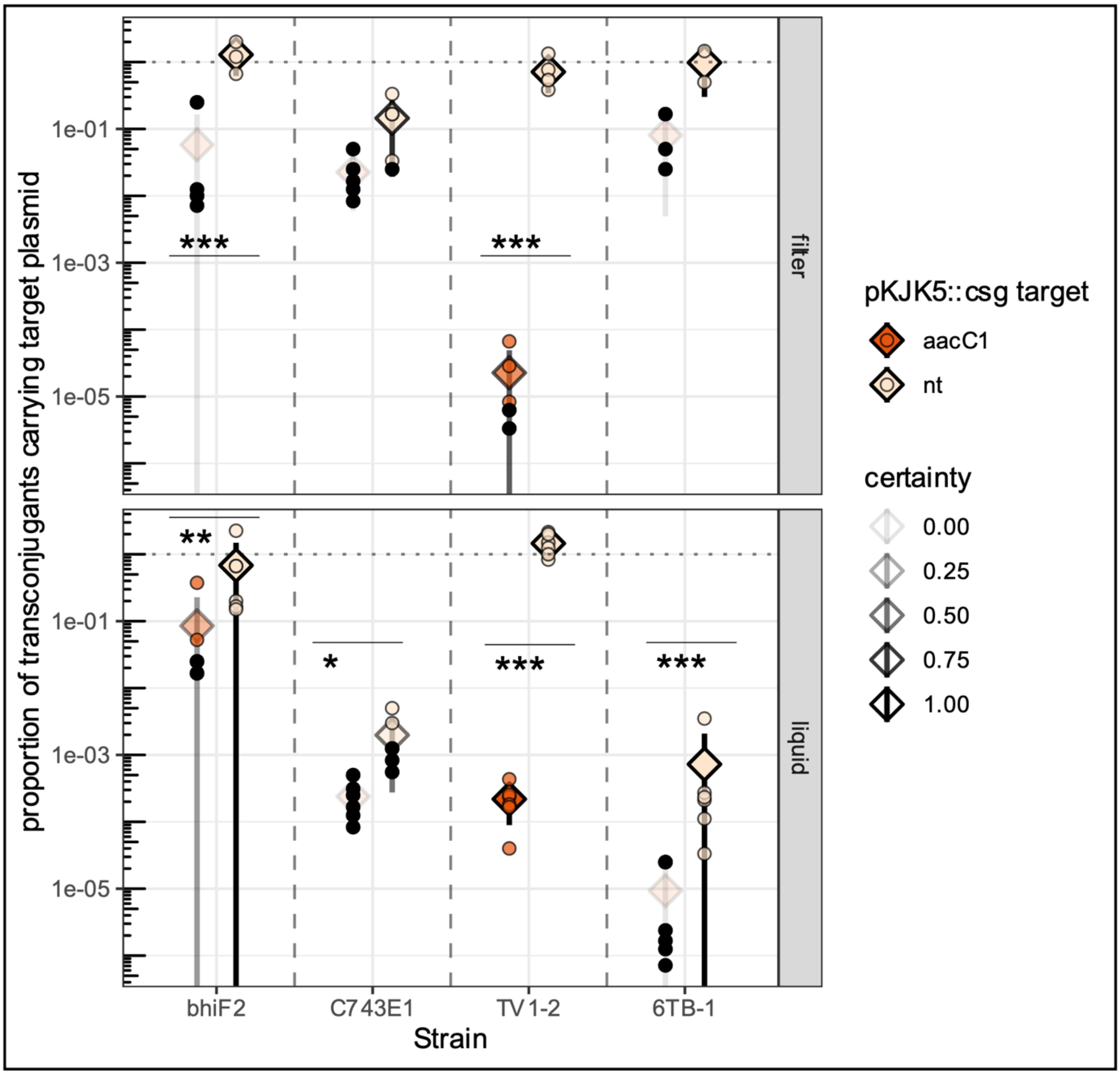
proportion of transconjugants carrying target plasmid
1e-01
1e-03
1e-05
***
***
**
*
***
***
filter
liquid
bhiF2
C743E1
TV1-2
6TB-1
Strain
pKJK5::csg target
aacC1
nt
certainty
0.00
0.25
0.50
0.75
1.00

65

66

Figure S4: Conjugation efficiency of pKJK5::csg to various isolates.

Means (diamonds) and standard deviation (lines) of recipients carrying pKJK5::csg divided by total recipients after delivery by solid-surface conjugative filter mating or by liquid mating using *E. coli* DH5α donors. N=5-6. The dotted line indicates 100%.

bhiF2, C743E1, TV1-2, 6TB-1: coliform pig faeces, environmental, and human isolates. SBW25: *Pseudomonas fluorescens* SBW25. These data are from the same dataset as presented in Figure S1.

Conjugation efficiency of pKJK5::csg to *Pseudomonas aeruginosa* PA14 was excluded from analysis due to some growth of recipient-only controls on pKJK5-selective plates.

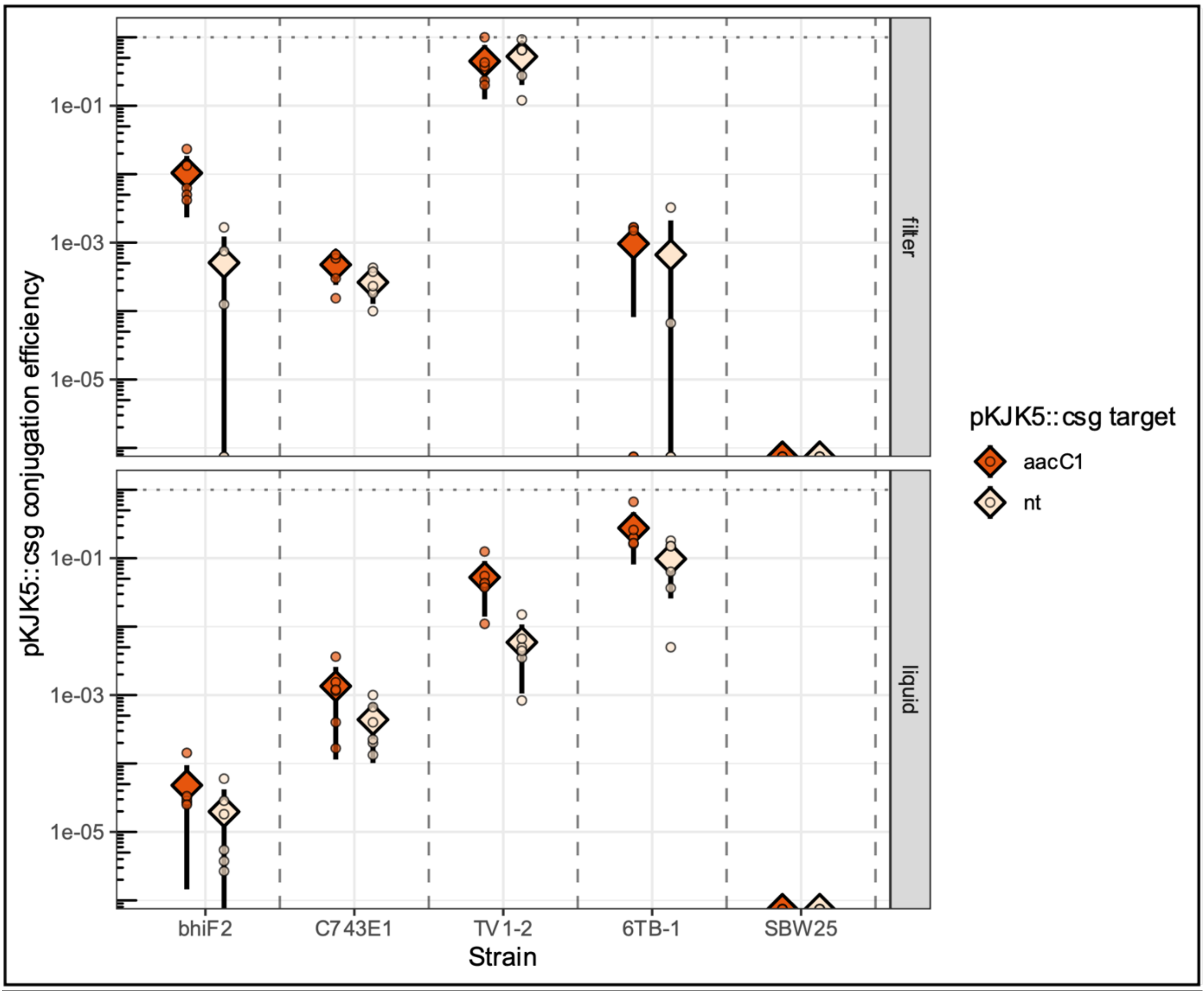

<u>**Table S1**: Isolate information.</u> Further information on the identity of isolates used in the broad host-range transformation experiment.

**Name**	**Source**	**Species**	**Other information**
bhiF2	Pig faeces samples	*Escherichia / Shigella*	Strain characterised by Sanger sequencing of 16S PCR only (this study).
C743E1	Human rectal swab	*Escherichia coli*	ST131; O16:H5. Strain characterised by PCR testing and Illumina sequencing (Leonard *et al.*, 2018; Leonard, in prep)
TV1-2	Sewage water	*Escherichia coli*	ST196; O8:H7. Strain characterised by PCR testing and Illumina sequencing (Leonard *et al.*, 2018; Leonard, in prep)
6TB-1	Bathing water	*Escherichia coli*	ST527; O139:H9. Strain characterised by PCR testing and Illumina sequencing (Leonard *et al.*, 2018; Leonard, in prep)
PA14	Laboratory strain	*Pseudomonas aeruginosa*	Originally isolated from burns patient in the 1970s (Schroth *et al.*, 2018)
SBW25	Laboratory strain	*Pseudomonas fluorescens*	Originally isolated from sugar beet in the 1990s (De Leij *et al.*, 1995)

References:

De Leij, F., Sutton, E.J., Whipps, J.M., Fenlon, J.S. and Lynch, J.M. 1995. Impact of Field Release of Genetically Modified Pseudomonas fluorescens on Indigenous Microbial Populations of Wheat. *Applied and Environmental Microbiology* 61: 3443–3453.

Leonard, A. in prep.

Leonard, A.F.C., Zhang, L., Balfour, A.J., Garside, R., Hawkey, P.M., Murray, A.K., Ukoumunne, O.C. and Gaze, W.H. 2018. Exposure to and colonisation by antibiotic-resistant E. coli in UK coastal water users: Environmental surveillance, exposure assessment, and epidemiological study (Beach Bum Survey). *Environment International* 1–8.

Schroth, M.N., Cho, J.J., Green, S.K., Kominos, S.D. and Microbiology Society PublishingYR 2018 2018. Epidemiology of Pseudomonas aeruginosa in agricultural areas*. *Journal of Medical Microbiology* 67: 1191–1201.

Table S2 – CRISPR targeting failure during plasmid removal experiment

Colony counts [cfu/mL] of samples on different selective media after the target plasmid removal experiment (Figure 3B). Selective media: recipients with pKJK5 and pHERD30T – KTG; recipients with pKJK5 – KT. CRISPR targeting failure incidence is calculated as the proportion of recipients with pKJK5 which also contain pHERD30T, and in the non-targeting control simply refers to the proportions of transconjugants which have not lost pHERD30T due to segregation. Full raw data can be found as supplementary data.

	**pKJK5::csg[aacC1] treatment**			**pKJK5::csg[nt] non-targeting control**		
replicate	**Recipients with pKJK5 and pHERD30T [cfu/mL]**	**Recipients with pKJK5 [cfu/mL]**	**CRISPR targeting failure incidence [% of cases]**	**Recipients with pKJK5 and pHERD30T [cfu/mL]**	**Recipients with pKJK5 [cfu/mL]**	**CRISPR targeting failure incidence [% of cases]**
1	6×10^2	2.6×10^6	0.023%	5.4×10^6	1.46×10^7	36.99%
2	4.6×10^2	7.2×10^6	0.0064%	7.0×10^6	1.24×10^7	56.45%
3	1.7×10^3	3.6×10^6	0.047%	9.0×10^6	2.92×10^7	30.82%
4	3.4×10^2	2.0×10^6	0.017%	1.32×10^7	2.78×10^7	47.48%
5	2×10^1	3.0×10^6	0.00067%	1.16×10^7	2.44×10^7	47.54%
6	2.6×10^2	1.0×10^6	0.026%	7.0×10^6	2.66×10^7	26.32%
**mean**	**5.63×10^2**	**3.23×10^6**	**0.020%**	**8.87×10^6**	**2.25×10^7**	**40.93%**

Table S3 – Statistical model estimates and coefficients

Estimates and coefficients of each model listed in Methods.

<table>
<tr><td colspan="6">pKJK5::csg conjugative delivery: target plasmid retention (Figure 3A; “target plasmid”)</td></tr>
<tr><td colspan="6">glm(formula = proportion ~ mating + medium + mating:medium, family = binomial(link = "logit"), data = stats_df, weights = k_raw)
Explanatory variables: ‘mating’ – pKJK5::csg target; ‘medium’ – Plasmid selected for.</td></tr>
<tr><td>Weighted Residuals:</td><td></td><td></td><td></td><td></td><td></td></tr>
<tr><td></td><td>Min</td><td>1Q</td><td>Median</td><td>3Q</td><td>Max</td></tr>
<tr><td></td><td>-6.3231</td><td>-1.0146</td><td>0.0004</td><td>0.2764</td><td>8.7101</td></tr>
<tr><td>Coefficients:</td><td></td><td></td><td></td><td></td><td></td></tr>
<tr><td></td><td>Estimate</td><td>Std. Error</td><td>t value</td><td>Pr(>|t|)</td><td></td></tr>
<tr><td>(Intercept)</td><td>0.3947</td><td>0.4406</td><td>0.896</td><td>0.3758</td><td></td></tr>
<tr><td>matingnt</td><td>0.2479</td><td>0.4744</td><td>0.523</td><td>0.6041</td><td></td></tr>
<tr><td>mediumkg</td><td>-1.3853</td><td>0.6562</td><td>-2.111</td><td>0.0411</td><td>*</td></tr>
<tr><td>mediumtkg</td><td>-9.5119</td><td>20.6322</td><td>-0.461</td><td>0.6473</td><td></td></tr>
<tr><td>mediumk</td><td>19.5396</td><td>2793.3829</td><td>0.007</td><td>0.9945</td><td></td></tr>
<tr><td>matingnt:mediumkg</td><td>0.9807</td><td>0.6999</td><td>1.401</td><td>0.1688</td><td></td></tr>
<tr><td>matingnt:mediumtkg</td><td>7.8143</td><td>20.6338</td><td>0.379</td><td>0.7069</td><td></td></tr>
<tr><td>matingnt:mediumk</td><td>1.6409</td><td>3939.154</td><td>0</td><td>0.9997</td><td></td></tr>
<tr><td colspan="6">Residual standard error: 2.681 on 40 degrees of freedom
Pseudo R-squared: 0.868
F-statistic: 0.3971 on 7 and 40 DF, p-value: 0.8984</td></tr>
<tr><td colspan="6">pKJK5::csg conjugative delivery: cell densities (Figure 3B)</td></tr>
<tr><td colspan="6">glm(formula = log(count) ~ mating * medium, family = gaussian(link = "identity"), data = stats2_df)
Explanatory variables: ‘mating’ – pKJK5::csg target; ‘medium’ – Selective medium</td></tr>
<tr><td>Residuals:</td><td></td><td></td><td></td><td></td><td></td></tr>
<tr><td></td><td>Min</td><td>1Q</td><td>Median</td><td>3Q</td><td>Max</td></tr>
<tr><td></td><td>-2.72958</td><td>-0.18964</td><td>0.00364</td><td>0.22975</td><td>1.71307</td></tr>
<tr><td>Coefficients:</td><td></td><td></td><td></td><td></td><td></td></tr>
<tr><td></td><td>Estimate</td><td>Std. Error</td><td>t value</td><td>Pr(>|t|)</td><td></td></tr>
<tr><td>(Intercept)</td><td>17.54014</td><td>0.23815</td><td>73.651</td><td>< 2e-16</td><td>***</td></tr>
<tr><td>matingut</td><td>-1.8089</td><td>0.3368</td><td>-5.371</td><td>1.34E-06</td><td>***</td></tr>
<tr><td>mediumt</td><td>0.07849</td><td>0.3368</td><td>0.233</td><td>0.817</td><td></td></tr>
<tr><td>mediumtk</td><td>-5.71999</td><td>0.3368</td><td>-16.984</td><td>< 2e-16</td><td>***</td></tr>
<tr><td>mediumkg</td><td>-6.68648</td><td>0.3368</td><td>-19.853</td><td>< 2e-16</td><td>***</td></tr>
<tr><td>mediumtkg</td><td>-14.81056</td><td>0.3368</td><td>-43.975</td><td>< 2e-16</td><td>***</td></tr>
<tr><td>mediumk</td><td>-5.15601</td><td>0.3368</td><td>-15.309</td><td>< 2e-16</td><td>***</td></tr>
<tr><td>matingut:mediumt</td><td>-0.08062</td><td>0.4763</td><td>-0.169</td><td>0.866</td><td></td></tr>
<tr><td>matingut:mediumtk</td><td>3.87076</td><td>0.4763</td><td>8.127</td><td>2.98E-11</td><td>***</td></tr>
<tr><td>matingut:mediumkg</td><td>4.69313</td><td>0.4763</td><td>9.853</td><td>3.73E-14</td><td>***</td></tr>
<tr><td>matingut:mediumtkg</td><td>12.03354</td><td>0.4763</td><td>25.265</td><td>< 2e-16</td><td>***</td></tr>
<tr><td>matingut:mediumk</td><td>3.75683</td><td>0.4763</td><td>7.887</td><td>7.62E-11</td><td>***</td></tr>
<tr><td colspan="6">Residual standard error: 0.5833 on 60 degrees of freedom
Multiple R-squared: 0.9805, Adjusted R-squared: 0.9769
F-statistic: 273.9 on 11 and 60 DF, p-value: < 2.2e-16</td></tr>
<tr><td colspan="6">pKJK5::csg prevents transformation in various host backgrounds (Figure 4)</td></tr>
<tr><td colspan="6">glm(formula = log(trans_eff) ~ Strain * Plasmid, family = inverse.gaussian(link = "log"), data = df_red)
Explanatory variables: ‘Plasmid’ – pKJK5::csg target.</td></tr>
<tr><td>Weighted Residuals:</td><td></td><td></td><td></td><td></td><td></td></tr>
<tr><td></td><td>Min</td><td>1Q</td><td>Median</td><td>3Q</td><td>Max</td></tr>
<tr><td></td><td>-0.67143</td><td>-0.01653</td><td>0</td><td>0.03523</td><td>1.22129</td></tr>
<tr><td>Coefficients:</td><td></td><td></td><td></td><td></td><td></td></tr>
<tr><td></td><td>Estimate</td><td>Std. Error</td><td>t value</td><td>Pr(>|t|)</td><td></td></tr>
<tr><td>(Intercept)</td><td>-1.11E+00</td><td>5.88E-02</td><td>-18.918</td><td>< 2e-16</td><td>***</td></tr>
<tr><td>StrainC743E1</td><td>-4.52E-16</td><td>9.61E-02</td><td>0</td><td>1.00E+00</td><td></td></tr>
<tr><td>StrainTV1-2</td><td>-2.69E-16</td><td>8.32E-02</td><td>0</td><td>1</td><td></td></tr>
<tr><td>Strain6TB-1</td><td>1.08E+00</td><td>1.17E-01</td><td>9.224</td><td>3.33E-12</td><td>***</td></tr>
<tr><td>StrainPA14</td><td>-1.32E-16</td><td>9.61E-02</td><td>0</td><td>1</td><td></td></tr>
<tr><td>StrainSBW25</td><td>2.26E+00</td><td>2.12E-01</td><td>10.66</td><td>3.00E-14</td><td>***</td></tr>
<tr><td>Plasmidnt</td><td>2.94E+00</td><td>2.41E-01</td><td>12.201</td><td>2.56E-16</td><td>***</td></tr>
<tr><td>StrainC743E1:Plasmidnt</td><td>1.46E-01</td><td>3.57E-01</td><td>0.409</td><td>0.6846</td><td></td></tr>
<tr><td>StrainTV1-2:Plasmidnt</td><td>1.75E-01</td><td>3.56E-01</td><td>0.491</td><td>6.26E-01</td><td></td></tr>
<tr><td>Strain6TB-1:Plasmidnt</td><td>-8.65E-01</td><td>3.69E-01</td><td>-2.348</td><td>2.31E-02</td><td>*</td></tr>
<tr><td>StrainPA14:Plasmidnt</td><td>2.50E-01</td><td>3.85E-01</td><td>0.649</td><td>0.5192</td><td></td></tr>
<tr><td>StrainSBW25:Plasmidnt</td><td>-2.11E+00</td><td>4.04E-01</td><td>-5.232</td><td>3.64E-06</td><td>***</td></tr>
<tr><td colspan="6">Residual standard error: 0.2296 on 48 degrees of freedom
Multiple R-squared: 0.9885, Adjusted R-squared: 0.9858</td></tr>
</table>

F-statistic: 374.6 on 11 and 48 DF, p-value: < 2.2e-16					
Target plasmid proportions after conjugative delivery of pKJK5::csg to various isolates (Figure S1) – filter mating					
glm(formula = proportions ~ pKJK5_type + recipient + pKJK5_type:recipient, family = inverse.gaussian(link = "log"), data = filter(figs1_stats, mating_type == "filter"))					
Weighted Residuals:					
	Min	1Q	Median	3Q	Max
	-1.8269	-0.5751	-0.1756	0.4926	2.7823
Coefficients:					
	Estimate	Std. Error	t value	Pr(>|t|)	
(Intercept)	-0.00334	0.439104	-0.008	0.99396	
pKJK5_typent	-0.16621	0.59674	-0.279	0.78181	
recipientC743E1	-1.56911	0.482661	-3.251	0.00211	**
recipientTV1-2	-1.39262	0.490633	-2.838	0.00663	**
recipient6TB-1	0.371064	0.687205	0.54	0.59172	
recipientPA14	-0.5251	0.553949	-0.948	0.34792	
recipientSBW25	0.134506	0.642949	0.209	0.83518	
pKJK5_typent:recipientC743E1	-0.72636	0.642412	-1.131	0.26381	
pKJK5_typent:recipientTV1-2	1.113869	0.726341	1.534	0.13171	
pKJK5_typent:recipient6TB-1	-0.01642	0.931842	-0.018	0.98601	
pKJK5_typent:recipientPA14	-0.01968	0.751564	-0.026	0.97922	
pKJK5_typent:recipientSBW25	0.124506	0.887825	0.14	0.88906	
Residual standard error: 0.9835 on 48 degrees of freedom<br>Multiple R-squared: 0.3214, Adjusted R-squared: 0.1659<br>F-statistic: 2.067 on 11 and 48 DF, p-value: 0.04185					
Target plasmid proportions after conjugative delivery of pKJK5::csg to various isolates (Figure S1) – liquid mating					
glm(formula = proportions ~ pKJK5_type + recipient + pKJK5_type:recipient, family = inverse.gaussian(link = "log"), data = filter(figs1_stats, mating_type == "liquid"))					
Weighted Residuals:					
	Min	1Q	Median	3Q	Max
	-2.6690	-0.4359	-0.1263	0.3178	3.7052
Coefficients:					
	Estimate	Std. Error	t value	Pr(>|t|)	
(Intercept)	0.04456	0.42176	0.106	0.916	
pKJK5_typent	0.15093	0.62027	0.243	0.809	
recipientC743E1	-2.83917	0.43391	-6.543	1.50E-08	***
recipientTV1-2	0.14353	0.61904	0.232	0.817	
recipient6TB-1	0.4026	0.66628	0.604	0.548	
recipientPA14	-0.28829	0.55786	-0.517	0.607	
recipientSBW25	0.18073	0.6253	0.289	0.774	
pKJK5_typent:recipientC743E1	0.07659	0.6389	0.12	0.905	
pKJK5_typent:recipientTV1-2	-0.41349	0.86486	-0.478	0.634	
pKJK5_typent:recipient6TB-1	-0.72845	0.8945	-0.814	0.419	
pKJK5_typent:recipientPA14	-0.27254	0.79758	-0.342	0.734	
pKJK5_typent:recipientSBW25	-0.57209	0.8589	-0.666	0.508	
Residual standard error: 1.01 on 60 degrees of freedom<br>Multiple R-squared: 0.403, Adjusted R-squared: 0.2935<br>F-statistic: 3.682 on 11 and 60 DF, p-value: 0.0005008					
TV1-2 recipients after pKJK5::csg delivery by filter mating (Figure S2)					
glm(formula = proportions ~ pKJK5_type + plasmid_content + pKJK5_type:plasmid_content, family = binomial(link = "log"), data = filter(fig5_stats, proportions != 1), weights = A_raw)					
Weighted Residuals:					
	Min	1Q	Median	3Q	Max
	-2.97387	-0.91653	-0.00529	0.53452	2.21072
Coefficients:					
	Estimate	Std. Error	t value	Pr(>|t|)	
(Intercept)	-1.4603	0.3908	-3.737	0.00108	**
pKJK5_typent	1.2192	0.4002	3.047	0.00573	**
plasmid_contentpKJK5	0.4387	0.5237	0.838	4.11E-01	
plasmid_contentboth	-9.7123	57.3102	-0.169	0.86691	
pKJK5_typent:plasmid_contentpKJK5	-0.6344	0.5445	-1.165	0.25591	
pKJK5_typent:plasmid_contentboth	8.963	57.3107	0.156	0.87709	
Residual standard error: 1.307 on 23 degrees of freedom<br>pseudo $R^2 = 0.707$<br>F-statistic: 1.164 on 5 and 23 DF, p-value: 0.3567					
Target plasmid proportions within transconjugants after conjugative delivery of pKJK5::Csg to various isolates (Figure S3): filter mating					

glm(formula = log(ATG_per_AT) ~ pKJK5_type + recipient + pKJK5_type:recipient, family = gaussian(link = "identity"), data = filter(figs2_stats, mating_type == "filter"))					
Residuals:					
	Min	1Q	Median	3Q	Max
	-1.3378	-0.6211	-0.1751	0.5627	2.5978
Coefficients:					
	Estimate	Std. Error	t value	Pr(>|t|)	
(Intercept)	-3.98406	0.450306	-8.847	3.57E-09	***
pKJK5_typent	4.140729	0.735346	5.631	7.36E-06	***
recipientC743E1	-0.00563	0.636828	-0.009	9.93E-01	
recipientTV1-2	-7.28966	0.636828	-11.447	1.96E-11	***
recipient6TB-1	1.158604	0.735346	1.576	0.1277	
pKJK5_typent:recipientC743E1	-2.50548	0.972771	-2.576	0.0163	*
pKJK5_typent:recipientTV1-2	6.701922	0.972771	6.89	3.20E-07	***
pKJK5_typent:recipient6TB-1	-1.4721	1.177128	-1.251	0.2227	
Residual standard error: 1.007 on 25 degrees of freedom<br>Multiple R-squared: 0.9433, Adjusted R-squared: 0.9274<br>F-statistic: 59.42 on 7 and 25 DF, p-value: 5.277e-14					
Target plasmid proportions within transconjugants after conjugative delivery of pKJK5::Csg to various isolates (Figure S3): liquid mating					
glm(formula = log(ATG_per_AT) ~ pKJK5_type + recipient + pKJK5_type:recipient, family = gaussian(link = "identity"), data = filter(figs2_stats, mating_type == "liquid"))					
Residuals:					
	Min	1Q	Median	3Q	Max
	-1.9581	-0.7333	-0.1044	0.4388	2.6958
Coefficients:					
	Estimate	Std. Error	t value	Pr(>|t|)	
(Intercept)	-3.2486	0.4371	-7.432	4.72E-09	***
pKJK5_typent	2.3656	0.6181	3.827	4.46E-04	***
recipientC743E1	-5.2589	0.6181	-8.508	1.64E-10	***
recipientTV1-2	-5.3866	0.6181	-8.714	8.73E-11	***
recipient6TB-1	-9.2831	0.6181	-15.018	< 2e-16	***
pKJK5_typent:recipientC743E1	-0.3684	0.8742	-0.421	0.675688	
pKJK5_typent:recipientTV1-2	6.5866	0.8742	7.535	3.41E-09	***
pKJK5_typent:recipient6TB-1	1.8153	0.8742	2.077	0.044302	*
Residual standard error: 1.071 on 40 degrees of freedom<br>Multiple R-squared: 0.9465, Adjusted R-squared: 0.9372<br>F-statistic: 101.2 on 7 and 40 DF, p-value: < 2.2e-16					
